# Optimal Duration of Fluorouracil-Based Adjuvant Chemotherapy for Patients with Resectable Gastric Cancer

**DOI:** 10.1371/journal.pone.0083196

**Published:** 2013-12-26

**Authors:** Jing-lei Qu, Xin Li, Xiu-juan Qu, Zhi-tu Zhu, Li-zhong Zhou, Yue-e Teng, Jing-dong Zhang, Bo Jin, Ming-fang Zhao, Ping Yu, Yun-peng Liu

**Affiliations:** 1 Department of Medical Oncology, The First Hospital of China Medical University, Shenyang, China; 2 Department of Oncology, The First Affiliated Hospital of Liaoning Medical University, Jinzhou, China; 3 Department of Medical Oncology, The Fourth Hospital of Anshan, Anshan, China; University Hospital Heidelberg, Germany

## Abstract

**Background:**

Although several clinical trials have suggested that postoperative adjuvant chemotherapy can improve survival of patients with gastric cancer, the optimal treatment duration has not been studied. This retrospective analysis evaluated the outcomes of patients with gastric cancer treated with six cycles of fluorouracil-based treatment compared with a cohort treated with four or eight cycles.

**Methods:**

We retrospectively identified 237 patients with stage IB–IIIC gastric cancer who received four, six, or eight cycles of fluorouracil-based adjuvant chemotherapy administered every 3 weeks after radical gastrectomy. The endpoint was overall survival (OS). Factors associated with prognosis were also analyzed.

**Results:**

The estimated 3-year OS rates for the four-, six-, and eight-cycle cohorts were 54.4%, 76.1%, and 68.9%, respectively; and the estimated 5-year OS rates were 41.2%, 74.0%, and 65.8%, respectively. Patients who received six cycles were more likely to have a better OS than those who received four cycles (*P* = 0.002). Eight cycles failed to show an additional survival benefit (*P* = 0.454). In the multivariate analysis, the number of chemotherapy cycles was associated with OS independent of clinical covariates (*P*<0.05). Subgroup analysis suggested that among patients in all age groups examined, male patients, and subgroups of fluorouracil plus oxaliplatin combined chemotherapy, stage III, poor differentiation, and gastrectomy with D2 lymphadenectomy, six cycles of adjuvant chemotherapy were associated with a statistically significant benefit of OS compared with four cycles (*P*<0.05).

**Conclusions:**

Six cycles of adjuvant chemotherapy might lead to a favorable outcome for patients with gastric cancer, and two further cycles could not provide an additional clinical benefit.

## Introduction

Gastric cancer is the fourth most common malignancy and the second most common cause of cancer-related mortality worldwide [1]. Surgery is the only potentially curative therapy for gastric cancer. Despite R0 resection, an appreciable proportion of patients still experience disease relapse, and the 5-year survival rate is disappointing. Several clinical trials recently suggested that postoperative adjuvant chemotherapy can improve patient outcomes [2], [3]. One meta-analysis documented that chemotherapy resulted in an 18% reduction in the mortality hazard compared with surgery alone [4]. Therefore, postoperative adjuvant chemotherapy has gained acceptance in both Eastern and Western countries.

There is no worldwide consensus regarding the adjuvant chemotherapy regimen for gastric cancer, but treatments are always based on fluorouracil (FU). The current standard of care in Japan is daily administration of the oral FU derivative S-1 for 4 out of every 6 weeks for 1 year based on the ACTS-GC study [2]. Findings from the CLASSIC trial in which we participated support the use of eight cycles of capecitabine plus oxaliplatin administered every 3 weeks [3]. Northern Europe favors a perioperative approach of three cycles of a preoperative and postoperative ECF (epirubicin, cisplatin and fluorouracil) regimen based on the results of the MAGIC trial [5]. These studies showed a survival advantage associated with postoperative and perioperative chemotherapy compared with surgery alone, although the employed duration of treatment differed among them.

The goal of adjuvant chemotherapy is to eliminate micrometastatic disease to improve survival. Several recent studies have addressed the issue of optimal duration of treatment, which impacts the efficacy of chemotherapy and quality of life for patients [6]–[8]. An inadequate duration of chemotherapy would lead to an increased risk of recurrence, and prolonged chemotherapy cannot enhance the survival benefit, instead impairing the immune response as a result of cumulative toxicity. A retrospective study showed that among elderly patients with colon cancer who discontinued treatment early, mortality rates were nearly twice as high as those among patients who completed 5 to 7 months of treatment [6]. For women with relatively low-risk primary breast cancer, extending chemotherapy from four to six cycles did not improve clinical outcomes [7]. In the adjuvant setting of non-small cell lung cancer, orthodoxy has recommended limiting treatment to four cycles [8]. However, the optimal duration of adjuvant chemotherapy that maximizes the survival benefit in gastric cancer is unclear.

In our center, six or eight cycles of adjuvant postoperative chemotherapy administered every 3 weeks were recommended for patients with stage IB–IIIC gastric cancer according to clinicopathological features and performance status. However, in clinical practice, a proportion of patients failed to complete the planned chemotherapy because of personal willingness rather than a poor performance status or severe side effects of chemotherapy. Considering that no prospective randomized trials have addressed the optimal duration of adjuvant chemotherapy for gastric cancer, we performed a retrospective analysis to compare the effects of four, six, and eight cycles of FU-based treatment on overall survival (OS) in patients with gastric cancer.

## Methods

### Ethics Statement

This retrospective study was carried out by three cancer centers from the Liaoning Province of China (the First Hospital of China Medical University, the First Affiliated Hospital of Liaoning Medical University, and the Fourth Hospital of Anshan). The study was approved by the institutional review board of each institution. All the participants provided written informed consent before enrollment.

### Patients

All patients received adjuvant chemotherapy after radical gastrectomy with D1 or D2 lymphadenectomy and had histologically confirmed stage IB–IIIC gastric cancer according to the American Joint Committee on Cancer (AJCC) TNM Staging Classification for Carcinoma, Seventh Edition [9]. Other principal inclusion criteria were as follows: all patients received four, six, or eight cycles of FU-based adjuvant chemotherapy; received adjuvant chemotherapy within 3 months after surgery; had no double-cancer history; and had not received neoadjuvant chemotherapy or adjuvant radiation. Patients with gastroesophageal junction cancer and patients who were lost to follow-up or died within 6 months of diagnosis were excluded. Between June 2004 and February 2012, a total of 237 patients met the inclusion criteria and were analyzed in this study. Patients were assessed for toxicity after each cycle of treatment according to the National Cancer Institute Common Toxicity Criteria version 3.0. Performance status were monitored and evaluated before each cycle of treatment based on Eastern Cooperative Oncology Group Performance Status Scale (ECOG PS).

### Statistical Analysis

The primary analysis involved evaluation of the association between the number of cycles of chemotherapy and OS, which was calculated from the time of surgery until death or the last follow-up visit. Secondary analysis were 3 year disease-free survival (DFS), defined as the time from surgery to the time of recurrence and safety (hematologic and gastrointestinal toxicities). Patient characteristics at diagnosis were compared by the number of cycles indicated, and chi-square tests were used to determine the significance of differences. Survival analysis was performed by the Kaplan-Meier method, and differences were assessed by the two-tailed log-rank test. To evaluate the impact of the number of treatment cycles on OS, univariate and multivariate analyses using a Cox proportional hazard regression model were carried out, and hazard ratios (HR) were estimated with 95% confidence interval (95% CI) limits. Multivariate analysis was performed by a forward stepwise addition with removal of covariates found to be associated with survival in univariate models (*P*<0.10). A two-sided significance test with a *P* value of <0.05 was considered to be statistically significant. Statistical analysis was carried out using SPSS 16.0 (Statistical Package for the Social Sciences; SPSS Inc., Chicago, IL, USA).

## Results

### Characteristics

Patient characteristics are listed in Table 1. Characteristics were well balanced between study arms with the exception of age at diagnosis (*P* = 0.048). Patients were administered four, six, or eight cycles of FU monochemotherapy, FU plus oxaliplatin, or other FU-based chemotherapy combinations, and each cycle lasted 21 days. Of 237 patients enrolled in the study, 67 were treated with four cycles, 105 were treated with six cycles, and 65 were treated with eight cycles. In all groups combined, the median age was 57 years (range, 30–76 years) with 172 male and 65 female patients. Sixty percent of patients had stage III disease, 62% had T4 tumors, 26% had more than six lymph node metastases (N3), 70% had poorly differentiated tumors, and 72% underwent gastrectomy with D2 lymph node dissection. Among patients treated with FU plus oxaliplatin, 6% had stage IB disease, 33% had stage II disease, 18% had stage IIIA disease, 19% had stage IIIB disease, and 24% had stage IIIC disease.

**Table 1:** Patient characteristics according to number of adjuvant chemotherapy cycles.

Characteristics	All (*n* = 237)	4 Cycles (*n* = 67)	6 Cycles (*n* = 105)	8 Cycles (*n* = 65)
	*n* (%)	*n* (%)	*n* (%)	*n* (%)
Age at diagnosis (median, range)	57 (30–76)	58 (36–75)	57 (30–73)	54 (30–76)
<60	148 (62.4)	34 (50.7)	68 (64.8)	46 (70.8)
≥60	89 (37.5)	33 (49.3)	37(35.2)	19 (29.2)
Gender				
Male	172 (72.6)	52 (77.6)	72 (68.6)	48 (73.8)
Female	65 (27.4)	15 (22.4)	33 (31.4)	17 (26.2)
Performance status (ECOG)				
0	126 (53.2)	36 (53.7)	54 (51.4)	36 (55.4)
1	111 (46.8)	31 (46.3)	51 (48.6)	29 (44.6)
Chemotherapy regimen				
FU monochemotheray	24 (10.1)	8 (11.9)	8 (7.6)	8 (12.3)
FU plus oxaliplatin	145 (61.2)	42 (62.7)	69 (78.1)	34 (52.3)
Other FU-based combinations	68 (28.7)	17 (28.4)	28 (26.7)	23 (35.4)
AJCC stage				
IB	13 (5.5)	4 (6.0)	6 (5.7)	3 (4.6)
II	81 (34.2)	23 (34.3)	40 (38.1)	18 (27.7)
IIIA	43 (18.1)	11 (16.4)	18 (17.1)	14 (21.5)
IIIB	44 (18.6)	11 (16.4)	17 (16.2)	16 (24.6)
IIIC	56 (23.6)	18 (26.9)	24 (22.9)	14 (21.5)
Tumor classification				
T1	9 (3.8)	1 (1.5)	7 (6.7)	1 (1.5)
T2	32 (13.5)	10 (14.9)	13 (12.4)	9 (13.8)
T3	49 (20.7)	10 (14.9)	22 (21.0)	17 (26.2)
T4	147 (62.0)	46 (68.7)	63 (60.0)	38 (58.5)
Nodal classification				
N0	64 (27.0)	22 (32.8)	25 (23.8)	17 (26.2)
N1	52 (21.9)	12 (17.9)	28 (26.7)	12 (18.5)
N2	59 (24.9)	14 (20.9)	26 (24.8)	19 (29.2)
N3	62 (26.2)	19 (28.4)	26 (24.8)	17 (26.2)
Histology grade				
G1–G2	72 (30.4)	17 (25.4)	31 (29.5)	24 (36.9)
G3–G4	165 (69.6)	50 (74.6)	74 (70.5)	41 (63.1)
Lymph node dissection				
D1	66 (27.8)	15 (22.4)	35 (33.3)	16 (24.6)
D2	171 (72.2)	52 (77.6)	70 (66.7)	49 (75.4)

Abbreviations: ECOG, Eastern Cooperative Oncology Group; FU, fluorouracil; AJCC, American Joint Committee on Cancer; G1, well differentiated; G2, moderately differentiated; G3, poorly differentiated; G4, undifferentiated.

### Survival Analysis

**Figure 1:**
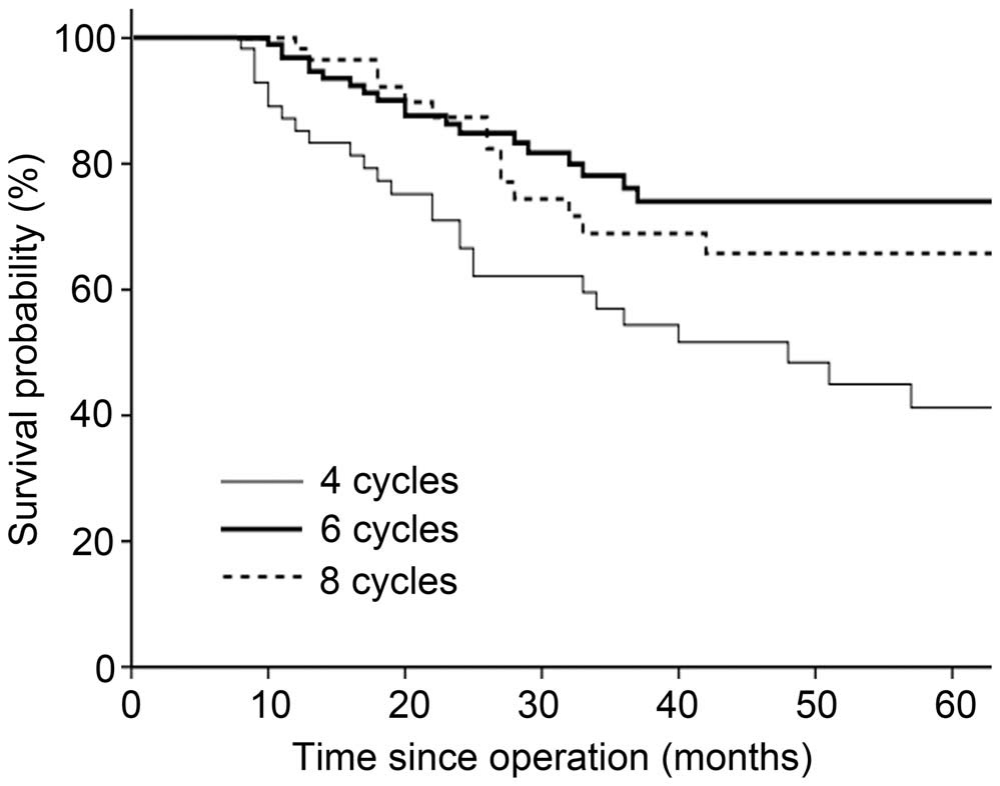
Kaplan-Meier survival curves by number of treatment cycles in all patients.

OS was analyzed in all 237 enrolled patients. With a median follow-up duration of 26 months (range, 6–99 months), none of the patients had evidence of disease progression at completing chemotherapy. The estimated 3-year OS rates for four, six, and eight cycles were 54.4%, 76.1%, and 68.9%, respectively; and the estimated 5-year OS rates were 41.2%, 74.0%, and 65.8%, respectively. Kaplan-Meier curves for these groups are illustrated in Figure 1. OS was better in patients who received six chemotherapy cycles than in those who received four cycles (*P* = 0.002). Compared with six cycles, two additional FU-based chemotherapy cycles did not improve OS (*P* = 0.454). The results of univariate analysis indicated that the number of chemotherapy cycles and stage had prognostic significance. In multivariate analysis, the number of chemotherapy cycles remained significantly independent of clinical covariates. The HRs for six and eight cycles compared with four cycles were 0.42 (95% CI 0.24–0.75, *P* = 0.004) and 0.51 (95% CI 0.27–0.97, *P* = 0.039), respectively (Table 2). Of 237 patients enrolled in the study, DFS was achieved in 108 patients (26 in the four-cycle group, 53 in the six-cycle group and 29 in the eight-cycle group). The 3-year DFS for four, six, and eight cycles were 40.5%, 74.5%, and 76.9%, respectively. Patients who received six cycles were have a better DFS than those who received four cycles (*P* = 0.009), and eight cycles failed to show an additional survival benefit (*P* = 0.618).

**Table 2:** Univariate and multivariate analysis for overall survival.

Factors	Univariate analysis			Multivariate analysis		
	HR	95% CI	*P* value	HR	95% CI	*P* value
Age at diagnosis (years)						
<60	1.00					
≥60	1.53	0.92–2.54	0.103			
Gender						
Male	1.00					
Female	1.45	0.85–2.48	0.177			
AJCC stage						
I+II	1.00			1.00		
III	3.68	1.91–7.07	<0.001	3.72	1.93–7.18	<0.001
Histology grade						
G1–G2	1.00			1.00		
G3–G4	2.01	0.99–4.09	0.053	1.85	0.90–3.77	0.092
Cycles of chemotherapy						
4	1.00			1.00		
6	0.41	0.23–0.73	0.002	0.42	0.24–0.75	0.004
8	0.51	0.27–0.96	0.037	0.51	0.27–0.97	0.039
Lymph node dissection						
D1	1.00					
D2	0.65	0.38–1.11	0.114			

Abbreviations: AJCC, American Joint Committee on Cancer; G1, well differentiated; G2, moderately differentiated; G3, poorly differentiated; G4, undifferentiated; HR, hazard ratio; CI, confidence interval.

### Subgroup Analysis

**Figure 2:**
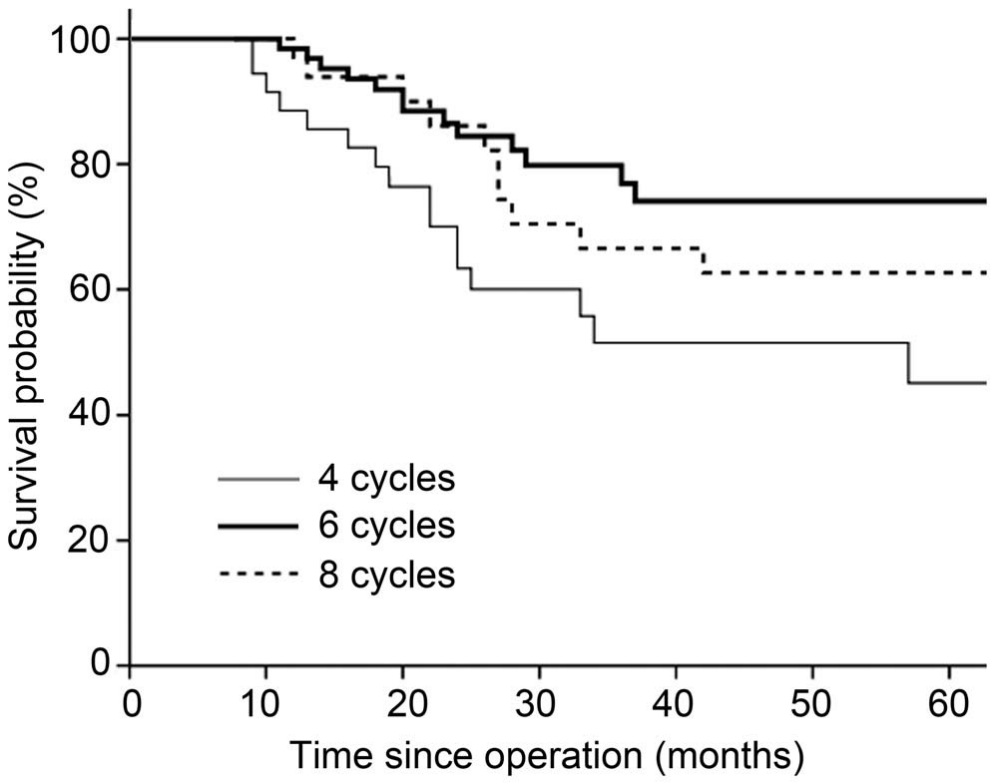
Kaplan-Meier survival curves by number of treatment cycles in the FU plus oxaliplatin subgroup.

Considering that the majority of patients (61%) received FU plus oxaliplatin combined chemotherapy, we evaluated the survival outcomes among these patients. Within this subset, patients who received six cycles had a better OS than those who received four cycles (HR 0.42, 95% CI 0.20–0.86, *P* = 0.017). Nevertheless, there was no benefit of eight cycles over six cycles (HR 1.49, 95% CI 0.68–3.29, *P* = 0.323). Figure 2 shows the OS of patients by treatment duration. The 3-year OS rates for the four-, six-, and eight-cycle cohorts were 51.4%, 76.9%, and 66.5%, respectively. However, in the subset of patients who received FU ± non-oxaliplatin therapy, six cycles produced a trend toward a better OS compared with four cycles (HR 0.39, 95% CI 0.14–1.07, *P* = 0.068), and there was no significant survival difference between six- and eight-cycle groups (HR 0.88, 95% CI 0.25–3.14, *P* = 0.846).

**Figure 3:**
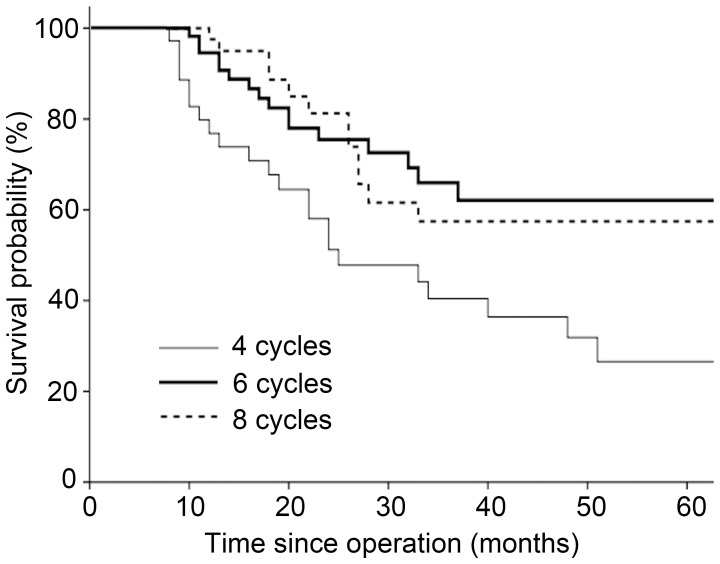
Kaplan-Meier survival curves by number of treatment cycles in patients with stage III cancer.

For the stage III subgroup, six treatment cycles was also associated with better survival compared with four cycles (HR 0.41, 95% CI 0.22–0.79, *P* = 0.007), and eight cycles was not superior to six cycles (HR 1.03, 95% CI 0.49–2.18, *P* = 0.939). The 3-year OS rates for the four-, six-, and eight-cycle cohorts were 40.4%, 66.0%, and 57.5%, respectively. Kaplan-Meier curves for these groups are illustrated in Figure 3.

**Figure 4:**
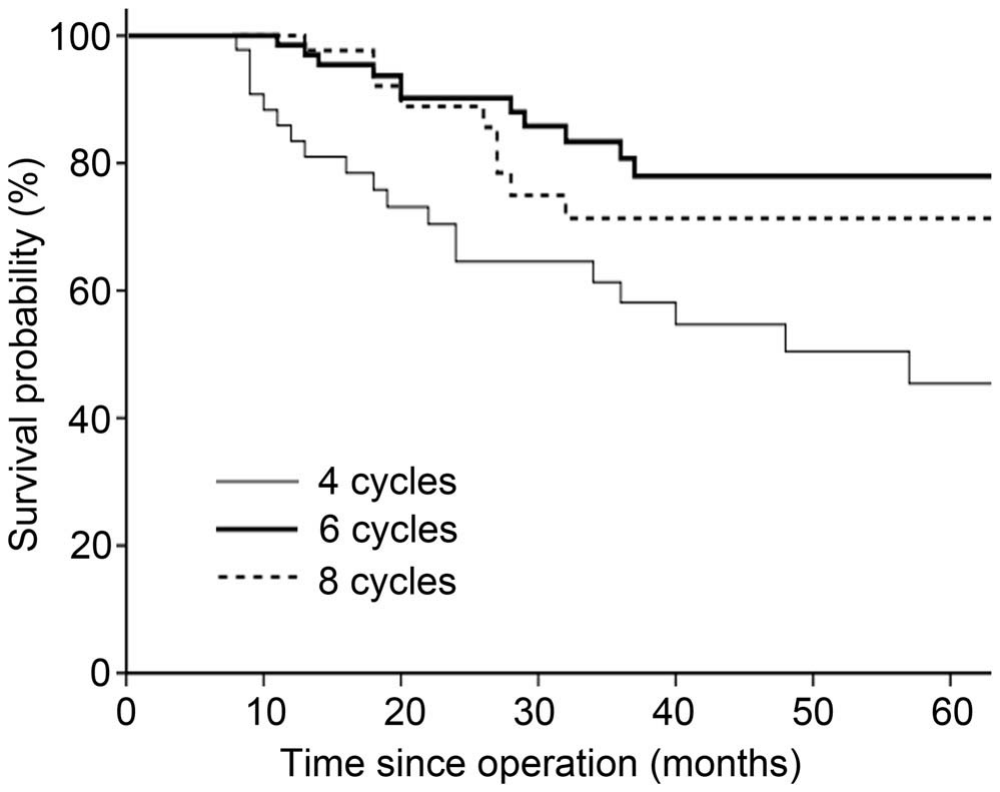
Kaplan-Meier survival curves by number of treatment cycles in patients who underwent D2 gastrectomy.

For patients who underwent D2 lymphadenectomy, six cycles were associated with an HR for mortality of 0.36 (95% CI 0.18–0.74, *P* = 0.006) versus four cycles, and eight cycles were associated with an HR for mortality of 1.41 (95% CI 0.61–3.28, *P* = 0.419) versus six cycles. Within this subset, the 3-year OS rates for the four-, six-, and eight-cycle cohorts were 58.1%, 80.7%, and 71.4%, respectively (Figure 4). However, for patients who underwent D1 lymphadenectomy, there was no significant survival advantage with six cycles compared with four cycles (*P* = 0.179) or eight cycles (*P* = 0.762).

**Figure 5:**
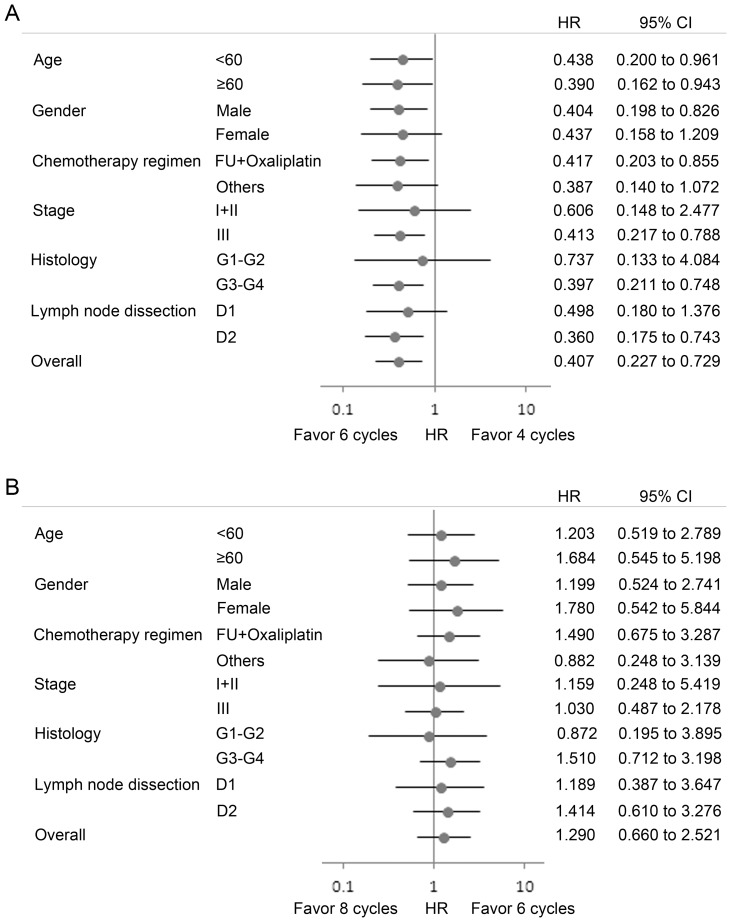
Hazard ratios (HRs) for death and 95% confidence intervals (CIs). In subgroup analyses, (A) six cycles of treatment were associated with an improved survival in most subgroups compared with four cycles, and (B) overall survival showed no significant difference between six and eight cycles.

Subgroup analysis was also undertaken according to age, gender, and histologic grade. Consistent with the overall patient population, the survival trend favored six over four cycles in most subgroups (Figure 5A), and the treatment effects of six and eight cycles showed no significant difference (Figure 5B).

### Adverse Events and Performance Status

Of 237 patients enrolled in the study, 18 were excluded from the safety population (4 in the four-cycle group, 9 in the six-cycle group and 5 in the eight-cycle group. The reasons for exclusion were absence of toxicity-related follow-up information. Hematologic and gastrointestinal toxicities were generally mild and there was no treatment-related death. The most common grade 3 adverse events during four cycles of treatment were neutropenia, anorexia, nausea, and vomiting (Table 3), and there were no significant difference among the three duration groups. Grade 3 neutropenia occurred in 5 (7.9%) patients in the four-cycle group, 8 (8.3%) patients in the six-cycle group, and 4 (6.7%) patients in the eight-cycle group. No adverse events of more than grade 3 occurred in any group during four cycles of treatment. Performance status were monitored and evaluated before each cycle of treatment, there were no significant difference among the three duration groups. At the fourth cycle of treatment, 3 (4.8%) patients had a performance status of 2 in the four-cycle group, 5 (5.2%) in the six-cycle group, and 3 (5.0%) in the eight-cycle group (Table 4).

**Table 3:** Adverse events observed during four cycles of treatment.

Adverse event	Grade 3, *n* (%)		
	4 Cycles (*n* = 63)	6 Cycles (*n* = 96)	8 Cycles (*n* = 60)
Neutropenia	5 (7.9)	8 (8.3)	4 (6.7)
Anorexia	3 (4.8)	3 (3.1)	2 (3.3)
Nausea	4 (6.3)	5 (5.2)	4 (6.7)
Vomiting	2 (3.2)	4 (4.2)	2 (3.3)

**Table 4:** Performance status evaluation at the fourth cycle of treatment.

Performance status (ECOG)	4 Cycles (*n* = 63)	6 Cycles (*n* = 96)	8 Cycles (*n* = 60)
	*n* (%)	*n* (%)	*n* (%)
0	18 (28.6)	28 (29.2)	19 (31.7)
1	42 (66.7)	63 (65.6)	38 (63.3)
2	3 (4.8)	5 (5.2)	3 (5.0)

Abbreviations: ECOG, Eastern Cooperative Oncology Group.

## Discussion

The issue of optimal duration of adjuvant treatment has been addressed for a variety of tumors, including colorectal, breast, non-small cell lung, and ovarian cancers [6]–[8], [10], [11]. However, there is still no consensus regarding the optimal duration of adjuvant chemotherapy for gastric cancer. Postoperative oral FU-based chemotherapy, such as S-1 for 1 year and capecitabine plus oxaliplatin for 6 months, is a proven effective treatment option for localized gastric cancer after D2 gastrectomy [2], [3]. Nevertheless, it is difficult to determine which regimen is superior because there is a lack of prospective trials comparing these regimens and durations with one another. Because of the relatively high recurrence rate of patients with gastric cancer, ethical issues will unlikely approve the evaluation of the optimal duration of adjuvant treatment in a prospective fashion. In this context, the present study offers some suggestions regarding the ideal duration of FU-based adjuvant chemotherapy for gastric cancer. The results suggests that six cycles of treatment are adequate, there is no additional benefit associated with eight cycles, and early termination of treatment is associated with worse overall mortality. Multivariate analysis showed that the number of chemotherapy cycles was an independent prognostic factor. In subgroup analysis, six cycles produced a significantly better or a trend toward a better OS compared with four cycles, as well as an OS similar to that of eight cycles in almost all subgroups.

In the subset of patients treated with FU plus oxaliplatin, the survival trend was consistent with that of the overall population. Within the subset, for patients with stage II–IIIB disease, the 3-year OS rates for four, six, and eight cycles were 66.6%, 86.0%, and 74.7%, respectively. These results were comparable with those of the CLASSIC trial, in which almost all patients had stage II–IIIB diaease (AJCC staging, Sixth Edition) and the 3-year OS rate was 83% in the capecitabine plus oxaliplatin group [3]. It seemed that shortening the treatment to six cycles in our study produced an efficacy similar to that of eight cycles in the CLASSIC study, although the different edition of staging used likely produced some differences. However, we need to bear in mind the safety of adjuvant treatment and patients' compliance. In the CLASSIC study, only 67% of patients assigned to the chemotherapy group completed eight cycles as planned, 56% who received chemotherapy had peripheral neuropathy, and 90% needed dose modifications because of adverse events [3]. The present study was limited by its inability to describe peripheral neuropathy. Nevertheless, given that oxaliplatin-induced peripheral neuropathy is a cumulative, dose-related toxic effect [12], it seems that the incidence and severity of toxicity might be reduced with a shorter duration of treatment. In view of the attenuating toxic effects and better compliance with shorter treatment durations, this retrospective study suggests that patients should be spared two additional toxic treatments with FU plus platinum without fear of compromising the disease outcome. In contrast, the FU ± non-oxaliplatin subset failed to show a significant correlation between OS and the number of chemotherapy cycles; this may have been due to the sample size limitation. In terms of FU monotherapy, the ACTS-GC study showed that 1-year treatment with S-1 reduced the risk of death by 33.1% compared with surgery alone [2]. Among the patients in the safety population who received S-1, only 65.8% continued treatment for 1 year, and 46.5% needed dose modifications. However, whether FU monotherapy given for a shorter duration is as effective as that given for 1 year is unclear because no studies have made this comparison. Therefore, the present results should be further validated among patients treated with FU monotherapy and FU-based non-oxaliplatin regimens.

In general, it is likely that patients with a high risk of relapse preferentially receive more cycles of treatment. However, the present study suggestes that patients with stage III disease do not get additional benefits from more than six cycles of therapy. It seems that six treatment cycles is adequate for tumors moderately sensitive to chemotherapy in an adjuvant setting. However, for patients with early-stage disease, there was no significant survival difference among the three duration groups. A recent study showed that postoperative adjuvant chemotherapy did not produce survival benefits for patients with stage II gastric cancer [13]. Therefore, adjuvant chemotherapy in stage II gastric cancer patients is still controversial. Randomized controlled clinical trials are needed to evaluate the effect of adjuvant chemotherapy and optimal number of treatment cycles for patients with early-stage gastric cancer.

Notably, the current analysis included patients who underwent D1 and D2 lymphadenectomy. Our results showed that six cycles of chemotherapy were superior for patients who underwent D2 gastrectomy, while for patients who underwent D1 gastrectomy, six cycles did not improve survival with statistical significance compared with four cycles. It has been found that the extent of regional lymphadenectomy has a major impact on the recurrence pattern in operable gastric cancer [14]. The incidence of locoregional recurrence was higher in patients who underwent D1 gastrectomy, and the addition of postoperative chemoradiotherapy could compensate for suboptimal surgical outcomes [15]. The Intergroup 0116 trial, in which 90% of patients underwent D0 or D1 lymph node dissection, demonstrated that postoperative chemoradiotherapy reduced recurrence and improved survival in patients with gastric cancer [16]. However, the recent results of the ARTIST trial showed that postoperative chemoradiotherapy did not significantly reduce recurrence in patients with D2 resected gastric cancer compared with chemotherapy alone [17]. Therefore, patients with a limited D1 resection seem to especially benefit from chemoradiotherapy after surgery, but not from chemotherapy alone.

Our results showed that patients aged ≥60 years had a survival trend similar to that of patients aged <60 years in different treatment groups, providing further evidence that age does not impact the efficacy of adjuvant therapy in gastric cancer [18], [19]. Female patients who received six cycles failed to show a significant survival benefit compared with those who received four and eight cycles, however, six cycles was associated with a trend toward better survival. Among female subgroups in the CLASSIC study, 3-year disease-free survival was not significantly improved with chemotherapy compared with surgery alone [3]. Females reportedly experienced more severe 5-FU-related toxicity than did men with colorectal cancer in a pooled analysis [20]. Whether this greater FU toxicity has a negative impact on survival among women should be evaluated in future studies.

In the present study, 28% of patients discontinued their treatment early and received four cycles of treatment. Although it was reported that chemotherapy-induced side effects and performance status could have impacted on treatment decisions and survival, our data showed the most common grade 3 hematologic and gastrointestinal toxicities and performance status during four cycles of treatment were not significant difference among the three duration groups. Therefore, toxicity and performance status do not account for much of the mortality in four-cycle cohort. Postoperative chemotherapy for gastric cancer had consistently lacked powerful evidence until two recent phase III studies (ACTS GC trial and CLASSIC trial) published. Some patients lacked confidence in efficacy of the chemotherapy and were unwilling to bear the medical expenses and side effects caused by chemotherapy, although no more than grade 3 toxicity occurred. Moreover, none of the patients had evidence of disease progression at completing chemotherapy, which could exclude the impact of disease progression on treatment decisions.

Our study has some limitations. First, this study is based on retrospective data and inability to provide complete DFS. However, it was reported that DFS was strongly correlated with OS at the individual level based on the GASTRIC data [21]. Therefore, our data about OS could also reflect the impact of chemotherapy duration on prognosis. Second, the patients received various chemotherapy regimens. The main reason was that no chemotherapy regimen had been considered as the standard recommendation until the result of CLASSIC study published in 2012. In the study, the proportion of patients who treated with FU plus non-oxaliplatin or FU monochemotherapy was small, which makes it difficult to stratify the patients for further analysis. However, 61% of patients received FU plus oxaliplatin chemotherapy; within this subset, the survival trend was consistent with that of the overall population. Third, the study was inability to describe peripheral neuropathy toxicity. In the CLASSIC study, grade 3 or 4 peripheral neuropathy occurred only in 2% of patients. It seems that oxaliplatin-induced peripheral neuropathy does not account for survival difference among the groups.

To our knowledge, the present study is the first to explore the optimal duration of FU-based adjuvant chemotherapy in patients with gastric cancer. Given the lack of benefit of continuing treatment beyond six cycles in all patients and almost all subgroups, this retrospective analysis suggested that six cycles of FU-based adjuvant chemotherapy might achieve an efficacy platform with minimal toxicity in patients with gastric cancer. It is acknowledged that the subgroups were small and underpowered and that additional data are needed to establish the optimal number of cycles in future studies. Notwithstanding this, no definitive conclusions can be drawn until prospective randomized controlled trials are conducted. Considering the reduced incidences of toxicity and better quality of life with shorter durations of treatment, further studies addressing this issue are warranted.

## References

[1] Van Cutsem E, Dicato M, Geva R, Arber N, Bang Y, et al (2011) The diagnosis and management of gastric cancer: expert discussion and recommendations from the 12th ESMO/World Congress on Gastrointestinal Cancer, Barcelona, 2010. Ann Oncol 22 Suppl 5: v1–9. 21633049 10.1093/annonc/mdr284

[2] Sasako M, Sakuramoto S, Katai H, Kinoshita T, Furukawa H, et al (2011) Five-year outcomes of a randomized phase III trial comparing adjuvant chemotherapy with S-1 versus surgery alone in stage II or III gastric cancer. J Clin Oncol 29: 4387–4393. 22010012 10.1200/JCO.2011.36.5908

[3] Bang YJ, Kim YW, Yang HK, Chung HC, Park YK, et al (2012) Adjuvant capecitabine and oxaliplatin for gastric cancer after D2 gastrectomy (CLASSIC): a phase 3 open-label, randomised controlled trial. Lancet 379: 315–321. 22226517 10.1016/S0140-6736(11)61873-4

[4] GASTRIC (Global Advanced/Adjuvant Stomach Tumor Research International Collaboration) Group (2010) Paoletti X, Oba K, Burzykowski T, Michiels S, et al (2010) Benefit of adjuvant chemotherapy for resectable gastric cancer: a meta-analysis. JAMA 303: 1729–1737. 20442389 10.1001/jama.2010.534

[5] Cunningham D, Allum WH, Stenning SP, Thompson JN, Van de Velde CJ, et al (2006) Perioperative chemotherapy versus surgery alone for resectable gastroesophageal cancer. N Engl J Med 355: 11–20. 16822992 10.1056/NEJMoa055531

[6] Neugut AI, Matasar M, Wang X, McBride R, Jacobson JS, et al (2006) Duration of adjuvant chemotherapy for colon cancer and survival among the elderly. J Clin Oncol 24: 2368–2375. 16618946 10.1200/JCO.2005.04.5005

[7] Shulman LN, Cirrincione CT, Berry DA, Becker HP, Perez EA, et al (2012) Six cycles of doxorubicin and cyclophosphamide or Paclitaxel are not superior to four cycles as adjuvant chemotherapy for breast cancer in women with zero to three positive axillary nodes: Cancer and Leukemia Group B 40101. J Clin Oncol 30: 4071–4076. 22826271 10.1200/JCO.2011.40.6405 PMC3494835

[8] Socinski MA, Stinchcombe TE (2007) Duration of first-line chemotherapy in advanced non small-cell lung cancer: less is more in the era of effective subsequent therapies. J Clin Oncol 25: 5155–5157. 18024862 10.1200/JCO.2007.13.4015

[9] Edge SB, Byrd DR, Compton CC, Fritz AG, Greene FL, et al.. (2009) American Joint Committee on Cancer Staging Manual. 7th ed. New York: Springer.

[10] Des Guetz G, Uzzan B, Morere JF, Perret G, Nicolas P (2010) Duration of adjuvant chemotherapy for patients with non-metastatic colorectal cancer. Cochrane Database Syst Rev 1: CD007046. 10.1002/14651858.CD007046.pub2 PMC10632948 20091614

[11] Dizon DS, Weitzen S, Rojan A, Schwartz J, Miller J, et al (2006) Two for good measure: six versus eight cycles of carboplatin and paclitaxel as adjuvant treatment for epithelial ovarian cancer. Gynecol Oncol 100: 417–421. 16336992 10.1016/j.ygyno.2005.10.031

[12] Bennett BK, Park SB, Lin CS, Friedlander ML, Kiernan MC, et al (2012) Impact of oxaliplatin-induced neuropathy: a patient perspective. Support Care Cancer 20: 2959–2967. 22426503 10.1007/s00520-012-1428-5

[13] Chen S, Chen YB, Zhou ZW, Li W, Sun XW, et al (2011) No survival benefit from postoperative adjuvant chemotherapy after D2 radical resection for the patients with stage II gastric cancer. Am J Clin Oncol 34: 309–313. 20838324 10.1097/COC.0b013e3181dea94e

[14] Dikken JL, Jansen EP, Cats A, Bakker B, Hartgrink HH, et al (2010) Impact of the extent of surgery and postoperative chemoradiotherapy on recurrence patterns in gastric cancer. J Clin Oncol 28: 2430–2436. 20368551 10.1200/JCO.2009.26.9654

[15] Muratore A, Zimmitti G, Lo Tesoriere R, Mellano A, Massucco P, et al (2009) Low rates of loco-regional recurrence following extended lymph node dissection for gastric cancer. Eur J Surg Oncol 35: 588–592. 19162429 10.1016/j.ejso.2008.12.012

[16] Macdonald JS, Smalley SR, Benedetti J, Hundahl SA, Estes NC, et al (2001) Chemoradiotherapy after surgery compared with surgery alone for adenocarcinoma of the stomach or gastroesophageal junction. N Engl J Med 345: 725–730. 11547741 10.1056/NEJMoa010187

[17] Lee J, Lim do H, Kim S, Park SH, Park JO, et al (2012) Phase III trial comparing capecitabine plus cisplatin versus capecitabine plus cisplatin with concurrent capecitabine radiotherapy in completely resected gastric cancer with D2 lymph node dissection: the ARTIST trial. J Clin Oncol 30: 268–273. 22184384 10.1200/JCO.2011.39.1953

[18] Jin Y, Qiu MZ, Wang DS, Zhang DS, Ren C, et al (2013) Adjuvant chemotherapy for elderly patients with gastric cancer after D2 gastrectomy. PLoS One 8: e53149. 23359796 10.1371/journal.pone.0053149 PMC3554736

[19] Aoyama T, Yoshikawa T, Watanabe T, Hayashi T, Ogata T, et al (2012) Safety and feasibility of S-1 adjuvant chemotherapy for gastric cancer in elderly patients. Gastric Cancer 15: 76–82. 21717091 10.1007/s10120-011-0068-7

[20] Chansky K, Benedetti J, Macdonald JS (2005) Differences in toxicity between men and women treated with 5-fluorouracil therapy for colorectal carcinoma. Cancer 103: 1165–1171. 15693031 10.1002/cncr.20878

[21] Rougier P, Sakamoto J (2011) Surrogate endpoints for overall survival in resectable gastric cancer and in advanced gastric carcinoma: analysis of individual data from the GASTRIC collaboration. Ann Oncol 22 suppl 5: v10–18.

